# Altitude Measurement-Based Optimization of the Landing Process of UAVs

**DOI:** 10.3390/s21041151

**Published:** 2021-02-06

**Authors:** Dariusz Horla, Wojciech Giernacki, Jacek Cieślak, Pascual Campoy

**Affiliations:** 1 Poznan University of Technology, Faculty of Automatic Control, Robotics and Electrical Engineering, Institute of Robotics and Machine Intelligence, ul. Piotrowo 3a, 60-965 Poznan, Poland; wojciech.giernacki@put.poznan.pl (W.G.); jacek.cieslak95@gmail.com (J.C.); 2 ITTI Sp. z o.o., ul. Rubież 46, 62-612 Poznan, Poland; 3 Computer Vision and Aerial Robotics Group (CVAR) at Centre for Automation and Robotics (CAR), Universidad Politecnica Madrid (UPM), 28006 Madrid, Spain; pascual.campoy@upm.es

**Keywords:** optimization, energy, UAV, landing

## Abstract

The paper addresses the loop shaping problem in the altitude control of an unmanned aerial vehicle to land the flying robot with a specific landing scenario adopted. The proposed solution is optimal, in the sense of the selected performance indices, namely minimum-time, minimum-energy, and velocity-penalized related functions, achieving their minimal values, with numerous experiments conducted throughout the development and preparation to the Mohamed Bin Zayed International Robotics Challenge (MBZIRC 2020). A novel approach to generation of a reference altitude trajectory is presented, which is then tracked in a standard, though optimized, control loop. Three landing scenarios are considered, namely: minimum-time, minimum-energy, and velocity-penalized landing scenarios. The experimental results obtained with the use of the Simulink Support Package for Parrot Minidrones, and the OptiTrack motion capture system proved the effectiveness of the proposed approach.

## 1. Introduction

### 1.1. Motivation

Autonomous landing feature is a major component in fully autonomous unmanned aerial vehicles (UAVs), as it is the precision of landing what is important in autonomous docking of any UAV platform, as stated in [1], into recharging stations in missions requiring repeated flight operations, pick and place tasks, and in executing repetitive behavior of the UAVs. Let alone, it is a difficult task, and gets even more complicated when the target landing location is moving, as UAVs are recently used to deliver different types of loads in dense environments such as cities, due to their manoeuvrability and possibility of vertical take-off and landing (VTOL) [2,3]. Whenever the flying platform carries a load, or flies without any additional load attached, it usually suffers from the limited time of operation, and must land from time to time—either to have its batteries recharged or replaced. In a usual manner, the landing requires a vertical maneuver to take place, which almost always is neither optimized, nor optimal [4]. The optimization of the landing process is of prime importance whenever this stage of flight is repeated numerous times during the mission, as in the already-mentioned pick and place tasks. One should note several papers related to the landing problem on a static platform, related to landing algorithms, visual servoing techniques applied to landing on a specific area, both simulated and deployed on real drones. However, none of the references has demonstrated a successful application to a real UAV focused primarily on the optimized landing procedure taking energy or landing time issues into account.

This problem has been indirectly tackled out by the organizers of the Mohamed Bin Zayed International Robotics Challenge (MBZIRC), organized by the Khalifa University in Abu Dhabi, both in its first edition (pick and place/treasure hunt task), as well in 2020 edition (wall assembly task). It is also visible in the next, 2023, edition in marine application task. The tasks selected in 2020 challenge required the participating teams to put all efforts to push the borders of knowledge and develop new techniques of operation. This paper focuses on Challenge 2, where automatic landing is repeated numerous times, to place bricks in a wall-like shape in precise locations. This problematic issue has been mirrored by the scores obtained from the judges, especially in the Grand Challenge, where the teams were allowed to perform the approach once only, in which the Skyeye Team ranked in the third place, despite the windy conditions, and high outside temperature, affecting the electronic equipment. The surrounding area of the flying arena has been full of metal objects and metal constructions, which severely impeded the correct work of magnetometers.

**Figure 1:**
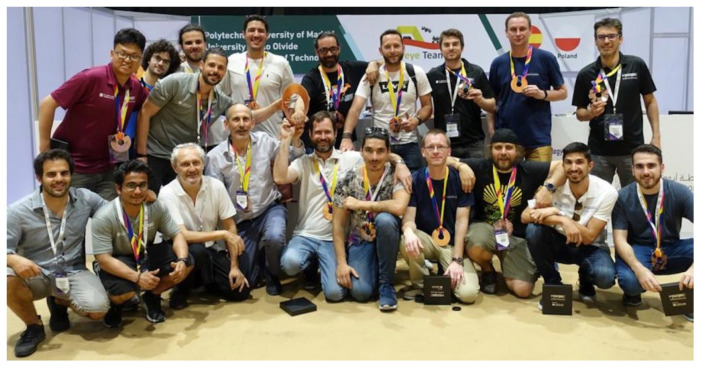
The Skyeye team award presentation.

The experimental platform used to perform the experiments referred to in this paper was the off-the-shelf UAV, Parrot Rolling Spider, selected to streamline the possible reproduction of the obtained results by the readers. During the MBZIRC’s Grand Challenge a different, far larger, platform was used and the Skyeye team scored the third place in this challenge, see Figure 1. Skyeye was a multi-disciplinary and multi-national team, in which every group from the Universidad Politecnica Madrid (CVAR-UPM), Pablo de Olavide University (SRL-UPO), and Poznan University of Technology (AeroLab) provided its best capabilities and experience to conquer during the MBZIRC, combining their strengths with the team of Dr. Antonio Franchi, Laboratory for Analysis and Architecture of Systems (CNRS-LAAS).

The major components of the optimized landing approach from the control side are described in this paper, where the core calculations to obtain optimal landing trajectories have been presented in the paper [5], and now, the complete experimental results are presented, to enable deployment of optimal procedure of landing UAVs by other researchers.

### 1.2. State of the Art Approaches

An increased popularity of UAVs was observed in the recent years, what is connected to their deployment areas, in which the common struggle to improve control algorithms that govern behaviour of UAVs can be easily identified, what is related to their reliability and safety of operation. As mentioned earlier, a spectrum of tasks carried out by UAVs demands a great deal of work from the side of control engineers responsible for controller tuning, which are commonly expected to enable the UAVs to perform agile and precise maneuvers [6], including precise landing, especially in the autonomous mode in real-world conditions.

This has been of great interest for researchers, though usually connected to landing vs. positioning problems, see [7,8] and a survey in [9] of vision-based autonomous landing approaches, or [10]. Autonomous return to the base position problem is tackled out in [11], with the use of the reinforcement learning techniques [12], what can be cast to the problem of path planning of the UAV landing on a moving target, as discussed in [13], where an iterative approach to the optimization problem is adopted. This can be extended to swarms of UAVs, as in [14], or to other applications [15]. Perched landing approach is addressed in [16] what states the problem is also valid for fixed-wing UAVs [17].

Vision-based techniques, as in [18], to estimate marker locations, intercepted UAV states, or landing platform location are quite commonly used, especially with attention paid to robustifying approaches, as in [19]. The approaches to the landing problem, available in the literature, as in [20,21,22] or [23], do not focus on optimization of the landing task itself, but on building control laws to land the UAV in the autonomous mode based on, e.g., detected markers. Autonomous outdoor landing procedures using static marker tracking are proposed in [24,25,26].

During the optimized landing process, the UAV is expected to precisely follow a reference trajectory to enable efficient landing with respect to some performance index, and the landing procedure can be executed at any stage of the flight, as per environmental issues, or low battery state, and to execute the flying scenario on the basis of some state machine operations. Across the years, with the increasing computational powers of on-board processors, off-line approaches to optimization, soft-computing techniques and algorithms for performing optimal tuning emerged. As in this paper, they require some performance-related cost functions. In addition, they also require any information concerning the model of the plant, clearly on the contrary to the approach presented in this paper. Even fuzzy-logic-based [6] or neural network-based [27] approaches require models to work, to embed this knowledge for further optimization, within rule bases. The modern branch of biology-related approaches to optimization [28] appear attractive even in this context, though usually require long calculation times and simulation-based runs. These again require models. Some relation to these approaches to UAVs can be found in [29].

### 1.3. Contribution of the Paper

This paper presents a complete approach to tuning the altitude control system to achieve optimal performance for optimal landing trajectories and different landing scenarios generated in [5]. The results include minimum-time landing scenario (emergency landing), energy-optimal landing and velocity-penalized landing scenarios. This is a problem not addressed in the literature so far, where multiple approaches to landing trajectory optimization can be found, but which are usually related to moving target positions, or non-zero terminal horizontal velocities.

It presents a deployment of a complete solution to optimization of the landing process on a static target, focusing on control techniques and optimization approaches to carry on with the landing process, to achieve both optimal performance, and to gain from all benefits of changing the position in a closed-loop system, enabling full response capabilities to potential disturbances. In our experimental campaign, the UAV hovers at certain altitude and its task is to land changing its altitude only, maintaining no displacement in horizontal axes. The landing scenarios considered include the tripled mentioned in the previous paragraph to mimic different regimes under which the UAV is supposed to operate in. The solution is verified by means of experiments carried out in flight in laboratory conditions, feeding the actual position to the controller, but to precisely calculate the performance indices. The performance indices include penalizing terms for altitude, rate of change in altitude, or control effort, in order to capture the standard requirements with respect to the landing process, ensuring full safety of the operator, as well as of the equipment. As detection of a landing pattern in an image from a camera is a separate task, just as calculating the relative position of the UAV with respect to the landing side using, say, vision algorithms, the paper focuses on the optimization-based controlling approach in which objective function consists of several different types of performance indices. Position and velocity of the UAV are estimated in a global frame of reference using the OptiTrack motion capture system to evaluate the performance of the closed-loop system, though, as above, the UAV itself is not fed with this information, and its operation is based on the fusion of measurements from its onboard sensors such as the ultrasonic, accelerometer, gyroscope, and air pressure sensors. The Rolling Spider drone has also been equipped with a downward facing camera, to obtain visual feedback, with all the information combined with the Kalman-filtered estimates based on the physical measurements, as per uncertain measurements in the indoor environment.

The novelty of the presented approach is the inclusion of the optimization techniques into the state-of-the art control methods to enable the execution of the landing task in real-world experimental conditions. The proposed algorithms are portable, and can be deployed on any VTOL platform, allowing the optimal results to be obtained. The approach presented in the paper yields in repeatability of the landing task, which is a core factor in autonomous UAV missions, especially for repeating pick and place problems, and the other practical issues so much visible in the MBZIRC Challenges.

## 2. Simplified Mathematical Model of the Rolling Spider Quadrotor

In this section, a basic notation is explained on the basis of a mathematical model of the Rolling Spider quadrotor, to introduce the reader to it. By no means is this model necessary to conduct experimental tuning runs, nevertheless the understanding of the interaction between signals, makes the performance index-related discussion put in a good context. The model of the Parrot Rolling Spider quadrotor driven by DC motors requires the introduction of coordinate systems at first (see [5]), to obtain information about position and orientation of the UAV. In the North-East-Down (NED) convention, x and y axes point towards the motors 1 and 2, whereas the axis z points downwards. For ϕ as the roll angle (x axis), θ as the pitch angle (y axis), and ψ as the yaw angle (z axis), rotation matrices $R_{\mathrm{yaw}}$, $R_{\mathrm{pitch}}$ and $R_{\mathrm{roll}}$, to obtain the final transformation matrix $R_{W2B}$ between global and local coordinate system is given for s(α):=sin(α), c(α):=cos(α), as:

(1) $$\begin{array}{cc} R_{W2B} & =R_{\mathrm{yaw}}R_{\mathrm{pitch}}R_{\mathrm{roll}}=\\ \; & =\left[\begin{array}{ccc} c(\theta )c(\psi ) & c(\psi )s(\theta )s(\phi )-c(\theta )s(\psi ) & s(\phi )s(\psi )+c(\phi )c(\psi )s(\theta )\\ c(\theta )s(\psi ) & c(\phi )c(\psi )+s(\theta )s(\phi )s(\psi ) & c(\phi )s(\theta )s(\psi )-c(\psi )s(\phi )\\ -s(\theta ) & c(\theta )s(\phi ) & c(\theta )c(\phi ) \end{array}\right]. \end{array}$$

Similarly, local rotational speeds $\underline{\Omega }={\left[p,q,r\right]}^{T}$ can be expressed as Euler angle derivatives $\underline{\dot{\Theta }}={\left[\dot{\phi },\dot{\theta },\dot{\psi }\right]}^{T}$.

Based on the derivations given in [5], the acceleration vector in three axes of a local coordinate system of the UAV

(2) $$\underline{\dot{v}}=\left[\begin{array}{c} \ddot{x}\\ \ddot{y}\\ \ddot{z} \end{array}\right]=\frac{1}{m}\left[\begin{array}{c} -T(c(\phi )s(\theta )c(\psi )+s(\phi )s(\psi ))\\ -T(c(\phi )s(\theta )s(\psi )-s(\phi )c(\psi ))\\ mg-T(c(\phi )c(\theta )) \end{array}\right],$$

and the thrust T[N] generated by the propellers (m[kg] is a mass of the UAV, and $g=9.81\;\frac{m}{s^{2}}$ is the gravity force)

(3) $$\begin{array}{cc} K_{a} & =C_{t}\rho \left(h\right)Ar^{2}, \end{array}$$

(4) $$\begin{array}{cc} T & =K_{a}\sum_{i=1}^{n}\left(\omega_{i}^{2}\right), \end{array}$$

where $\omega_{i}\;\left[\frac{\mathrm{rad}}{s}\right]$ denotes the rotational speed of the *i*-th propeller, $K_{a}$ [kg·m] is the aerodynamical constant, $C_{t}$ refers to the thrust constant, $\rho \;[\frac{\mathrm{kg}}{m^{3}}]$ denotes air density, h[m] gives vertical distance between the center of mass (COM) of the UAV and mounting height of the propellers, $A\;[m^{2}]$ is propellers’ surface area, r[m] refers to the propeller’s radius, and *n* is the number of propellers.

The quadrotor (n=4) has six degrees of freedom, thus, in order to describe its state, 12 state variables are used [30],

(5) $$\begin{array}{cc} \underline{x} & ={\left[x,\;y,\;z,\;\phi ,\;\theta ,\;\psi ,\;\dot{x},\;\dot{y},\;\dot{z},\;p,\;q,\;r\right]}^{T}, \end{array}$$

with $x_{1}=\dot{z}$ and $x_{2}=z$ to be referred henceforth as the state variables introduces for the purpose of position change in z axis. In (5), the symbol $\underline{x}$ refers to a full state vector, whereas *x* and $\dot{x}$ are position and velocity of the UAV, respectively, in axis x, etc.

The analysis of the landing process requires the model to be further simplified. It is assumed that the UAV does not move in XY plane and keeps a horizontal alignment, thus without any loss of generality it can be assumed for a VTOL UAV that:

(6) $$x=\dot{x}=\ddot{x}=y=\dot{y}=\ddot{y}=0,$$

(7) $$\begin{array}{cc} \phi & =\theta =0. \end{array}$$

This assumption is a natural consequence of changing the position in one axis only, maintaining the horizontal displacement due to actions of fixed-parameter XY controllers. As all four motors have the same rotational speed, $T=4K_{a}\omega^{2}$ and $\ddot{z}=g-\frac{T}{m}=g-4\frac{K_{a}}{m}\omega^{2}$.

Having assumed that the air density does not vary during the experiment, with $\alpha :=4\cdot \frac{K_{a}}{m}$, $u:=\omega^{2}$, T=αu, and $\ddot{z}=\alpha u-g$, the simplified model of dynamics in the z axis

(8) $$\begin{array}{ccc} {\dot{x}}_{1} & = & \ddot{z}=\alpha u-g\;, \end{array}$$

(9) $$\begin{array}{ccc} {\dot{x}}_{2} & = & \dot{z}=x_{1} \end{array}$$

is obtained. For a complete derivation of the model, see [5]. As can be seen, the simplified model actually presents the relation between the thrust force, gravitational constant, and position-velocity interplay.

## 3. Methodology behind the Optimal Reference Trajectory Generation

### 3.1. Landing Procedures

As during the landing process the altitude decreases, the reference trajectory leading to the ground level accepts no overshoots, independently on the landing scenario adopted. In this paper, three different scenarios are considered, namely: emergency landing (minimum-time landing), minimum-energy landing or velocity-penalized landing (planned landing). In all the scenarios, the reference trajectory changes the reference altitude $h_{\mathrm{start}}\to h_{\mathrm{ground}}$, with $h_{\mathrm{start}}$ as the initial altitude at $t_{0}$, and $h_{\mathrm{ground}}$ being the ground level altitude.

As the notation with *z* denoting current altitude has been previously adopted:

(10) $$\begin{array}{cc} z(0) & =h_{\mathrm{start}}, \end{array}$$

(11) $$\begin{array}{cc} z(t_{f}) & =\dot{z}\left(0\right)=\dot{z}\left(t_{f}\right)=0, \end{array}$$

(12) $$\begin{array}{cc} z(t) & \geq 0, \end{array}$$

where z(t) denotes the position of the UAV in z axis. The generated trajectory should in principle mimic the desired behaviour of the UAV at the landing stage, and forms a natural input to the control system both while performing tuning, as well as during the landing process, as the reference signal.

### 3.2. Considered Reference Trajectory Profiles

Based on the derivations given in [5], and dynamic optimization [31], the minimum-time landing scenario is composed of two stages. In the first stage, the thrust is zeroed admitting to a maximum altitude decrease rate. In the second stage, a full thrust is used to mimic the braking stage. The landing thus undergoes the reference altitude change in stage-by-stage fashion, switched between at time instant $t_{s}$ and the UAV lands at $t_{f}$ where ($T_{\max}=\alpha u_{\max}$ is the maximum torque, resulting from control signal cut-off level):

(13) $$\begin{array}{cc} t_{s} & =\sqrt{\frac{2h_{\mathrm{start}}\left(T_{\max}-g\right)}{gT_{\max}}}, \end{array}$$

(14) $$\begin{array}{cc} t_{f} & =\sqrt{\frac{2h_{\mathrm{start}}T_{\max}}{g(T_{\max}-g)}}. \end{array}$$

On the basis of (13) and (14), it is possible to provide the analytical formula for the reference landing trajectory

(15) $$\begin{array}{c} z=x_{2}^{*}=\left\{\begin{array}{ccc} h_{\mathrm{start}}-\frac{1}{2}gt^{2} & \Leftrightarrow & 0\leq t<t_{s}\\ h_{\mathrm{start}}-\frac{1}{2}g{t_{s}}^{2}-gt_{s}\left(t-t_{s}\right)+\frac{1}{2}\left(T_{\max}-g\right){\left(t-t_{s}\right)}^{2} & \Leftrightarrow & t_{s}\leq t<t_{f}\\ 0 & \Leftrightarrow & t\geq t_{f} \end{array}.\right. \end{array}$$

In the case of minimum-energy landing (also termed as false-zero landing), where the to-be-optimized cost function comprises non-negative altitude and control-energy-related terms with a positive weighing factor β

(16) $$\begin{array}{cc} J & =\int_{0}^{t}\left(x_{2}+\beta u^{2}\right)dt, \end{array}$$

the landing is also splitted into two stages:

(17) $$\begin{array}{cc} x_{2}\left(t\right) & =\left\{\begin{array}{cc} f(t) & \;\Leftrightarrow \;0<t\leq \frac{\pi }{\psi }\\ 0 & \;\Leftrightarrow \;\frac{\pi }{\psi }<t \end{array}\right., \end{array}$$

where f(t) reduces the altitude between $h_{\mathrm{start}}\to 0$. To eliminate the impact of the overshoot on the landing phase, the ground level should be virtually raised so that the minimum of $x_{2}$ equals always zero, what explains the ’false zero’ name of the approach, giving:

(18) $$\begin{array}{c} \begin{array}{ccc} x_{1}\left(0\right)=0 & x_{1}\left(\frac{\pi }{\psi }\right)=0 & x_{1}\left(t_{f}\to \infty \right)=0.\\ x_{2}\left(0\right)=h_{\mathrm{start}} & x_{2}\left(\frac{\pi }{\psi }\right)=0 & x_{2}\left(t_{f}\to \infty \right)=h_{e} \end{array} \end{array}$$

As the obtained optimal altitude trajectory presents some natural overshoot, and its local extremum is reached when velocity reaches zero, it is natural to assume this time instant should correspond to the minimum admissible altitude, reaching which the thrust is cut off. The value of β is the function of the initial altitude only.

The optimal trajectory is

(19) $$\begin{array}{cc} \; & x_{2}^{*}\left(t\right)=\left\{\begin{array}{cc} h_{start}\left(e^{-\psi t}\left(1-\frac{h_{\mathrm{start}}}{\left(1+e^{\pi }\right)}\right)\left(c\left(\psi t\right)+s\left(\psi t\right)\right)+\left(1-\frac{h_{\mathrm{start}}}{\left(1+e^{\pi }\right)}\right)\right) & \;\Leftrightarrow \;0<t\leq \frac{\pi }{\psi }\\ 0 & \;\Leftrightarrow \;\frac{\pi }{\psi }<t \end{array}\right., \end{array}$$

and the procedure for obtaining β is given in [5].

The third scenario refers to velocity-penalized landing, where the sub-integral expressions relate to velocity penalization, and control effort penalization terms,

(20) $$J=\int_{0}^{t}\left({x_{1}}^{2}+\beta u^{2}\right)dt,$$

with

(21) $$\begin{array}{c} x_{1}^{*}\left(t\right)=h_{\mathrm{start}}\left(e^{-\psi t}\left(-\psi c(\gamma t)-\gamma s(\gamma t)\right)+\frac{\psi }{\gamma }e^{-\psi t}\left(-\psi s(\gamma t)+\gamma c(\gamma t)\right)\right), \end{array}$$

(22) $$\begin{array}{c} x_{2}^{*}\left(t\right)=h_{\mathrm{start}}e^{-\psi t}\left(c\left(\gamma t\right)+\frac{\psi }{\gamma }s\left(\gamma t\right)\right). \end{array}$$

As in the case of minimum-energy landing, β should also be optimized using, preferably, the same method.

### 3.3. Application of the Optimal Reference Trajectory

The optimal reference trajectories for the three considered landing scenarios are generated to be fed to the altitude control system, to follow them as precisely as possible. To accomplish this task, it is expected that the altitude control system should have the dynamics allowing it to mimic the profile of the reference trajectory. For a selected altitude controller, the problem reduces thus to selecting the altitude controller gains (or parameters) as to achieve optimal results. Using this control policy will ensure the UAV is under control at all times, and does not work in the open loop.

To implement this approach, the authors used a software package (Simulink Support Package for Parrot Minidrones) developed by the manufacturer of the Rolling Spider UAV, as well as the motion capture system to fully evaluate the performance of the adopted solution. The gains were tuned with the use of a novel rapid tuning method, based on Fibonacci-search algorithm, outlined in [32], to achieve optimal results using the data obtained from the motion capture system.

The behavior of the UAV was initially simulated using the Simulink Support Package for Parrot Minidrones, to implement, verify and test the optimization tools for in-flight conditions, based on the basic block diagram available from the Support Package.

The control system was modified by introducing the I term to the existing PD altitude controller, to obtain

(23) $$\begin{array}{cc} u(t) & =k_{P}e\left(t\right)+k_{I}\int e\left(t\right)dt+k_{D}\frac{de}{dt}, \end{array}$$

with the integral gain $k_{I}=0.01$ identified on the basis of multiple tests, as a compromise between maintaining hover capabilities and good transient behavior. The altitude controller gains, i.e., $k_{P}$ and $k_{D}$ were tuned using the Fibonacci-search algorithm, shortly characterized in the following Section. The potential XY displacements have been kept at their minimal levels, as ensured by the nominal Rolling Spider controllers. When using the proposed solution, it is recommended to conduct tuning in Z axis, and for other approaches, to perform consecutive tuning campaigns in roll/pitch, X-Y axes, and finally yaw, respectively.

The Parrot Rolling Spider is equipped with the inertial measurement unit (IMU), allowing one to obtain estimates of the position and orientation of the drone in a 3D space. One of the core assumptions when conducting real-world experiments is the duration of the experiment. On the basis of several trials (T=10s and T=12s long), composed of ‘take off’, ‘hover’ and ‘land’ stages (the latter executed at t=7s), it was fixed that the IMU-based measurements were biased by errors incrementing over time, thus it was welcomed to reduce the duration of experiments to evaluate the performance in a more accurate way. It was found advantageous to use a MoCap system instead of the IMU to get the correct readings to calculate performance-related indices, to perform tuning.

## 4. Experimental Hardware Platform

Basic pre-MBZIRC trials of the optimized landing procedures were carried out on the Rolling Spider UAV. In 2016, the Department and Aeronautics and Astronautics (AeroAstro) from Massachusetts Institute of Technology (MIT) designed *Simulink Support Package for Parrot Minidrones* as MATLAB’s add-on. The software streamlines designing, simulation and rapid testing of control algorithms using real UAV platforms. It is dedicated to a family of Rolling Spider and Mambo drones by Parrot, and can also be used to perform model-in-the-loop simulations, as well as real-world experiments.

**Figure 2:**
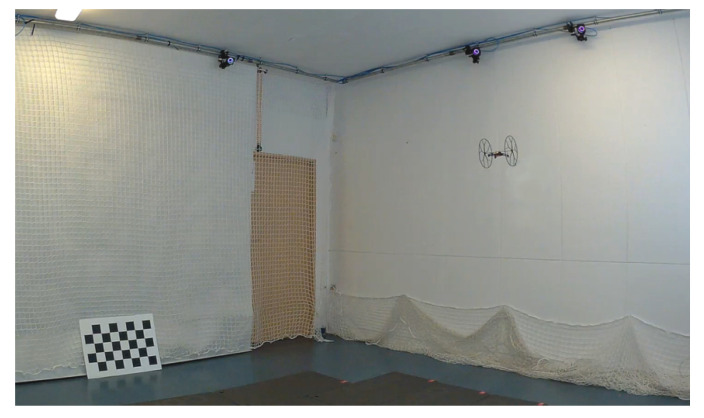
AeroLAB flying arena during the experimental flights.

To conduct the tuning experiment and the optimization of the landing process, it was necessary to use a reliable source of information concerning the position (esp. altitude) of the UAV. It was conducted with the use of the motion capture system, OptiTrack, composed of a set of cameras, and a dedicated software generating current position and orientation information of the markers on the basis of the visual data. The flying area was a room of a volume ca. 4×5×3m with a set of 10 OptiTrack cameras hung just below the ceiling (AeroLab, http://uav.put.poznan.pl), see Figure 2.

**Figure 3:**
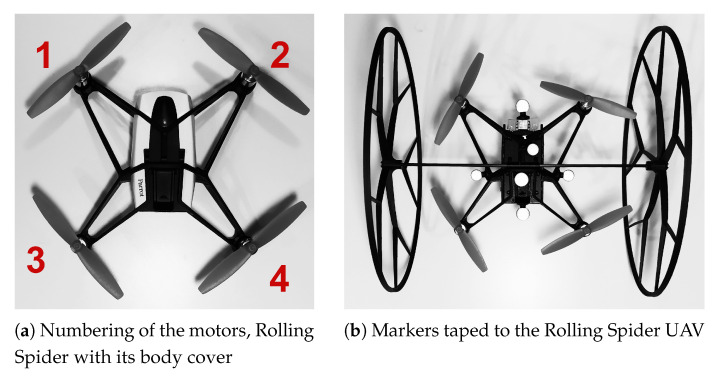
Experimental setup of the Rolling Spider.

A proper visibility of the UAV was ensured by placing markers on its body. Small sizes, and light weight of the Rolling Spider drone cause severe problems at the configuration stage, combined in addition with a small thrust force generated by the propellers. To conduct the experiments, all necessary parts of the body of the drone were removed, to compensate for the weight of the markers. The markers themselves were placed on the UAV with no symmetry, but still below the propellers’ level, in order not to disturb the air flow, nor to alter the dynamic properties of the structure. The placement of the markers is shown in Figure 3b.

During the experiments, the Rolling Spider was observed by a set of cameras positioning the markers taped to its body, what resulted in obtaining good position and orientation estimates. Still, some issues arose, connected to the decrease of the number of visible markers, occlusion of a marker by an element of the UAV, etc. The Appendix A gives further information on the topic.

**Figure 4:**
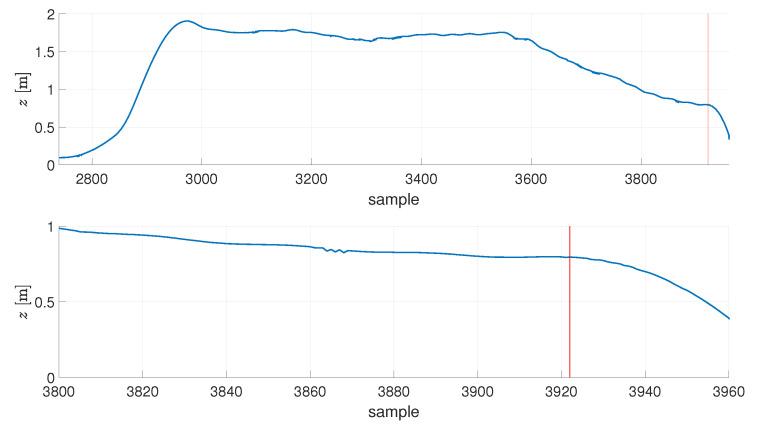
Marking of a sample terminating an experiment.

A proper calculation of the performance index for every flight required good synchronization between the data obtained from the OptiTrack and the reference signal fed to the Rolling Spider. As these two systems worked independently (and with different sampling frequencies), time stamp-based synchronization was impossible. The issue was solved by manual operation—after 10s of every experiment, the script running the optimization procedure terminated the experiment, and switched the DC motors of the UAV off. The OptiTrack continued the registration of the data, what enabled one to identify the time instant when the free fall stage began, see Figure 4. After identifying this time instant manually, the OptiTrack-based data was cropped to the length of 10s prior to the selected sample. Next, the reference signal values were calculated on the basis of analytical formulae (presented in the further part of the text) for every sample coming from the OptiTrack system.

**Figure 5:**
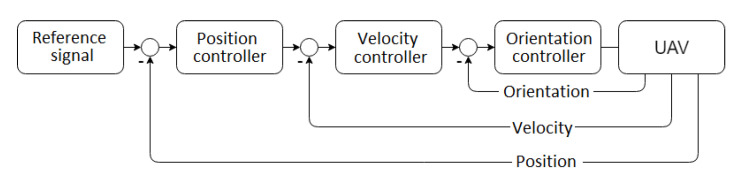
Typical cascade of controllers in UAVs.

As it is known, every multirotor-based UAV enables three basic control regimes: position control, velocity control and orientation control. In typical systems, a cascade of controllers takes on the regulation tasks [33], as depicted in Figure 5. In the current work, position control was used, thus no problems with pitch or roll stabilization occurred, as the presented cascaded structure of the control system obviously refers to control actions in XY, whereas here we are mainly occupied with change in z axis.

## 5. Altitude Controller Tuning Procedure

### 5.1. Short Characterization of the Rapid Tuning Algorithm

The gains of the altitude controller were found using the Fibonacci-search algorithm, which does not require any information concerning a model of the drone, but only calculating of some cost function is needed as the insight into the performance of the system. This cost function in control systems usually takes the form of a performance index, reflecting the true impact of the gains on the control system.

This adopted procedure is based on a simple one-dimensional zero-order algorithm, though by its use the method has multiple interesting features:

- the model of the UAV can be either unknown or imprecise;
- the value of the cost function used during the search is based on a performance index for a given time horizon, and can be obtained in a repetitive manner (by repeating consecutive experiments);
- the optimal gains of a controller are obtained in an iterative way.

Let a unimodal single-argument function F(x) be given in the following range of its argument: $\left[x^{\left(0^{-}\right)},\;x^{\left(0^{+}\right)}\right]$, with $x^{\left(0^{-}\right)}<x^{\left(0^{+}\right)}$. The information concerning current value of *F* can be obtained for any *x*, as it refers to execution of a single experiment. When the values of *F* at the two intermediate points are known, the following cases apply:

- $F\left(x^{\left(1^{-}\right)}\right)<F\left(x^{\left(1^{+}\right)}\right)$→ the minimum is in the range $\left[x^{\left(0^{-}\right)},\;x^{\left(1^{+}\right)}\right]$,
- $F\left(x^{\left(1^{-}\right)}\right)\geq F\left(x^{\left(1^{+}\right)}\right)$→ the minimum is in the range $\left[x^{\left(1^{-}\right)},\;x^{\left(0^{+}\right)}\right]$,
  where $x^{\left(i^{-}\right)}$ is the lower bound for the range $D^{\left(i\right)}$ at the *i*-th iteration, and $x^{\left(i^{+}\right)}$ is the upper bound for the sought parameter.

For the initial range of an argument $x\in D^{\left(0\right)}=\left[x^{\left(0^{-}\right)},\;x^{\left(0^{+}\right)}\right]$, search algorithm is as follows:

- evaluate the minimal number of iterations *N* for which the difference between true minimum $x^{*}$ and iterative solution ${\hat{x}}^{*}$ (it is assumed that it is in the middle of the range $D^{\left(N\right)}$) does not exceed the prescribed relative accuracy ϵ, where
  (24) $$|x^{*}-{\hat{x}}^{*}|\leq \epsilon \left(x^{\left(0^{+}\right)}-x^{\left(0^{-}\right)}\right)\;,$$
- for k=1,…,N:
  (1) select a pair of intermediate points ${\hat{x}}^{\left(k^{-}\right)}$, ${\hat{x}}^{\left(k^{+}\right)}$ (${\hat{x}}^{\left(k^{-}\right)}<{\hat{x}}^{\left(k^{+}\right)}$, $\left\{{\hat{x}}^{\left(k^{-}\right)},\;{\hat{x}}^{\left(k^{+}\right)}\right\}\in D^{\left(k-1\right)}$) from the range $D^{\left(k-1\right)}$;
  (2) obtain the new range $D^{\left(k\right)}$ evaluating its bounds as:
    (a) for $F\left({\hat{x}}^{\left(k^{-}\right)}\right)<F\left({\hat{x}}^{\left(k^{+}\right)}\right)$, $x^{\left(k+1\right)}\in D^{\left(k\right)}=\left[x^{\left(k-1^{-}\right)},\;{\hat{x}}^{\left(k^{+}\right)}\right]$;
    (b) for $F\left({\hat{x}}^{\left(k^{-}\right)}\right)\geq F\left({\hat{x}}^{\left(k^{+}\right)}\right)$, $x^{\left(k+1\right)}\in D^{\left(k\right)}=\left[{\hat{x}}^{\left(k^{-}\right)},\;x^{\left(k-1^{+}\right)}\right]$;
  (3) put k:=k+1;
- assume that ${\hat{x}}^{*}=\frac{1}{2}\;\left(x^{\left(N^{+}\right)}+x^{\left(N^{-}\right)}\right)$ is the solution to the problem.

To tune a pair of controller gains, as in the case of the altitude controller, with $k_{P}$ and $k_{D}$ tuned:

(1) calculate bounds on $k_{D}$ and $k_{P}$ for the given UAV, ensuring its stability;
(2) define initial value of $k_{P}^{\left(0\right)}$;
(3) using a sequence of Fibonacci-search iterations for the defined tolerance ϵ and k=0, implement the following bootstrapping technique (put $k_{P}^{\left(k+1\right)}=k_{P}^{\left(k\right)}$) until *N* iterations are performed:
  (3a) starting with the initial range for $k_{D}$ and fixed $k_{P}^{\left(k+1\right)}$, find by means of the Fibonacci-search method the optimal ${\hat{k}}_{D}^{*}\left(k+1\right)$, and proceed to the step 2b;
  (3b) starting with the initial range for $k_{P}$ and fixed $k_{D}^{\left(k+1\right)}={{\hat{k}}_{D}}^{*}\left(k+1\right)$, find by means of the Fibonacci-search method the optimal ${{\hat{k}}_{P}}^{*}\left(k+1\right)$; until the required number of bootstraps is not done, enter k:=k+1 and proceed to the step 2a.

It should be stressed that the number of iterations and requested number of bootstraps (in the paper equal to 2) imply a deterministic running time of the optimization algorithm, as the function of ϵ. The required number of iterations *N* to achieve selected accuracy ϵ satisfies $F_{N+1}\geq \frac{1}{\epsilon }$, and the two intermediate points are always selected as below [32]:

(25) $$\begin{array}{ccc} \;\;\;\;\;\;\;{\hat{x}}^{\left(k^{-}\right)} & = & x^{\left(k-1^{-}\right)}+\rho_{k}\left(x^{\left(k-1^{+}\right)}-x^{\left(k-1^{-}\right)}\right)\;, \end{array}$$

(26) $$\begin{array}{ccc} \;\;\;\;\;\;\;{\hat{x}}^{\left(k^{+}\right)} & = & x^{\left(k-1^{-}\right)}+\left(1-\rho_{k}\right)\left(x^{\left(k-1^{+}\right)}-x^{\left(k-1^{-}\right)}\right)\;, \end{array}$$

where

(27) $$\begin{array}{c} \rho_{k}=\frac{F_{N-k}}{F_{N-k+2}}\;, \end{array}$$

and $F_{j}$ denotes the *j*-th Fibonacci number.

The Fibonacci method approach to tuning was selected as a core of our tuning algorithm as per its properties. As is known, the contraction ration $\rho_{N}$ after *N* iterations is optimal, when compared to other zero-order approaches. The zero-order feature is of prime importance, while along with maintaining the minimal number of iterations to obtain the minimum with the epsilon tolerance, the overall computational complexity stays at a low level, avoiding estimation of gradients, Hessians, etc. At the same time, the difference between the pair of gains at the ends of the range is noticeable and allows the UAV to show visible difference in performance over a vast majority of iterations, in comparison, e.g., with a dichotomy zero-order method.

### 5.2. Tuning Issues

The choice of a performance index should mirror the performance of the closed-loop system, and its natural choice is was to use the integral of the absolute error index (IAE), defined for sampled-data systems as

(28) $$\begin{array}{cc} \mathrm{IAE} & =\sum_{i=1}^{n}\left|\epsilon_{i}\right|, \end{array}$$

(29) $$\begin{array}{cc} \epsilon_{i} & =z_{{\mathrm{ref}}_{i}}-z_{i}, \end{array}$$

where $\epsilon_{i}$ is the altitude tracking error, *n* denotes the number of samples taken into account, and the sampling period $T_{s}$ is a design parameter of the Rolling Spider UAV defined by the manufacturer and the developers of the Simulink Support Package for Parrot Minidrones, with $T_{s}=0.005\;s$.

The performance index (28) is incremented over time and puts a $L_{1}$ penalty on tracking errors, what is not fully proper for the optimization of the landing process, where any overshoot should be severely penalized (the UAV would fall below the ground level). Thus, the following modification to (28) was introduced:

(30) $$\begin{array}{cc} J & =\sum_{i=1}^{n}J_{i}=\sum_{i=1}^{n}k\left|\epsilon_{i}\right|,\\ k & =\left\{\begin{array}{cc} 1 & \Leftrightarrow z_{i}\geq h_{\mathrm{ground}}\\ p\in {ℛ}_{+} & \Leftrightarrow z_{i}<h_{\mathrm{ground}} \end{array}\right., \end{array}$$

where *p* was the weight for the negative tracking error. A good choice of this penalty parameter is the key factor for the tuning process.

On the basis of initial tests, and for the reference trajectories as in Section 3 modified by including an initial altitude signal of value $h_{\mathrm{start}}$ lasting for 4s to make the UAV take off and, subsequently, hover at the specific altitude, with

- $h_{\mathrm{start}}=2\;m$,
- $h_{\mathrm{ground}}=1\;m$,

it was fixed that p=30 results in a good capture of the performance, leading to:

(31) $$\begin{array}{cc} J & =\sum_{i=1}^{n}k\left|\epsilon_{i}\right|,\\ k & =\left\{\begin{array}{cc} 1 & \Leftrightarrow z_{i}\geq h_{\mathrm{ground}}\\ 30 & \Leftrightarrow z_{i}<h_{\mathrm{ground}}. \end{array}\right. \end{array}$$

In the considered Fibonacci-search algorithm, a pair of parameters is optimized in a bootstrap manner (one parameter is optimized, and the other one is kept constant and vice versa), streamlining the use of standard zero-order algorithms. A bootstrap comprises searching for the optimal values of a pair of arguments, and after two bootstraps it is agreed that the optimal tuning terminates.

For the optimization purposes, the cost function of the Fibonacci-search algorithm *F* is replaced by the performance index (31) measured in the horizon when the actual landing phase takes place, with the appropriate number of samples. In all the cases, when the optimization is carried out on the basis of simulations, it was assumed that: the initial value of $k_{P}=0.8$, the optimal gain $k_{P}$ lies in the range [1.5,7.0], $k_{D}$ lies in the range [0.1,2.5], and $\epsilon \leq 10^{-4}$ (N=19).

Naturally, the gains suitable for optimal landing are expected to cause severe oscillations during the take-off phase. This issue can be solved by changing the gains between those intended for take off, and the others for landing, with the special switching law. The abrupt change in gains would inevitably result in oscillations or even instability of the control loop, thus the step change between the gains can be smoothed by low-pass filtering the gains (take-off/landing switching) by the filter with the time constant of $T_{F}=1\;s$. By making a discrete-time model of this filter using the zero-order hold method, with $T_{s}=0.005\;s$, one gets:

(32) $$\begin{array}{cc} G_{F}\left(s\right) & =\frac{1}{s+1}, \end{array}$$

(33) $$\begin{array}{cc} G_{F}\left(z\right) & =\frac{0.004988}{z-0.995}. \end{array}$$

## 6. Tuning Based on the IMU Unit

**Figure 6:**
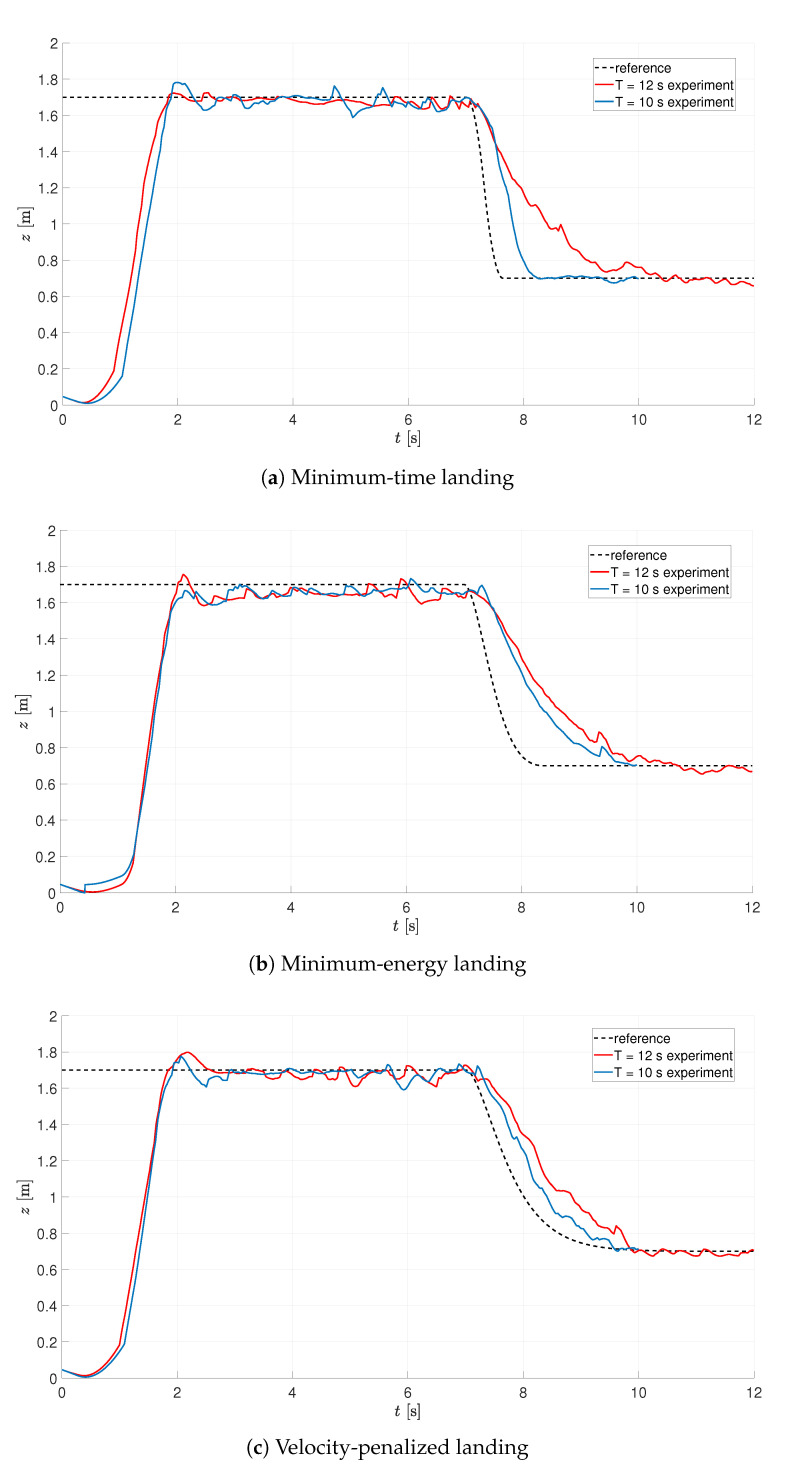
Optimization of landing on the basis of IMU readings (gains switched at t=3s).

The Parrot Rolling Spider UAV, as stated before, is equipped with the IMU unit, which gives estimates for position and orientation. The duration of the experiment is the major issue here, to avoid excessive accumulation of error over time. Two series of experiments were conducted in horizons of T=10s and T=12s, with the landing phase initiated at time instant t=7s, with the tuning results presented in Figure 6. In all the cases, when the optimization is carried out on the basis of IMU readings, it was assumed that: the initial value of $k_{P}=0.8$, the optimal gain $k_{P}$ lies in the range [0.1,7.0], $k_{D}$ is within [0.1,7.0], and ϵ≤0.05 (N=6).

The results of optimization for various landing scenarios, as well as the performance index values are presented in Table 1. Every tuning procedure was composed of two bootstrap sequences, in which the landing procedure was repeated time after time. A single collection of the performance index required the UAV to take off, hover, and when landing command was given at t=7s (gains changed at t=3s), the collection of samples to obtain the performance index values was initialized. In the first column, four stages for of the optimization procedure are listed, where stage 1 and 2 refers to the first bootstrap, and stage 3 and 4, to the second bootstrap. A bootstrap is composed of two tuning procedures (stages) executed one after another, where a single gain is tuned at a time, and the remaining one held constant on the value corresponding to the optimal one obtained either from the prior stage, or from the initial values. In columns 2–5 the final values of the tuned gains ($k_{P}$ and $k_{D}$) are listed, with the final value of the performance index *J*, for the 3 considered landing scenarios, and, in addition, for the take-off stage. As can be seen, the final values of *J* are minimized across the stages of optimization.

As can be seen from Figure 6, the performance of the system is better for a shorter horizon of the experiment, what is due to the impact of the environment on the UAV (external disturbances). The latter impedes the tuning procedure, as the values of the performance index are deteriorated along the way, what spoils the results of the optimization. From this reason, the remaining experiments were conducted in a shorter, 10-s horizon.

**Figure 7:**
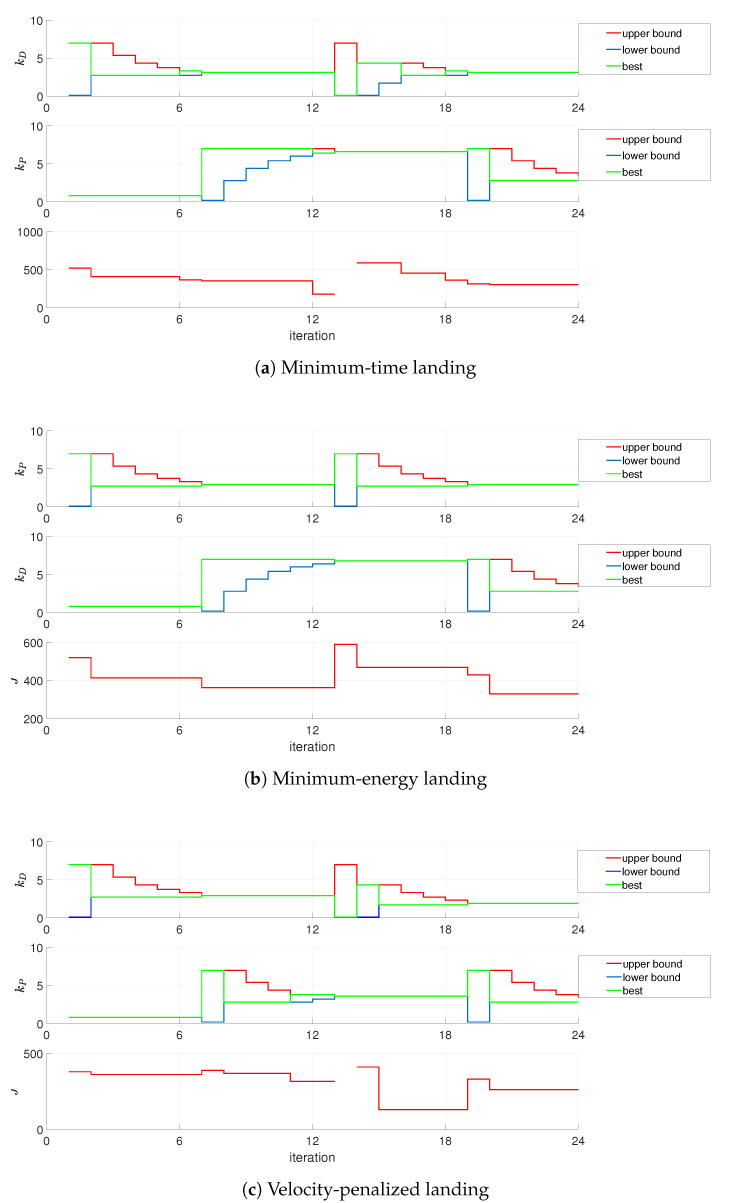
Optimization of landing on the basis of IMU readings—12s experiment (gain plots, missing *J* data denotes crash).

Consecutive stages of tuning, performance index values, as well as changes in gains $k_{P}$ and $k_{D}$ for the longer experiment conducted, are depicted in Figure 7 and presented in Table 2. Lack of performance index value at the *i*-th stage of the experiment refers to instability of the control loop for a particular configuration of gains, with the presumed value of the performance index set at J(i)=∞.

## 7. Tuning Based on the MoCap System

To give a proper evaluation of the tuning results, it was necessary to use a reliable source of position and orientation measurements. The data from the OptiTrack was used to evaluate performance index only, and not used to feed the position/orientation information back to the UAV, thus the basic flight properties of the Rolling Spider were not modified when comparing IMU and OT approaches.

**Figure 8:**
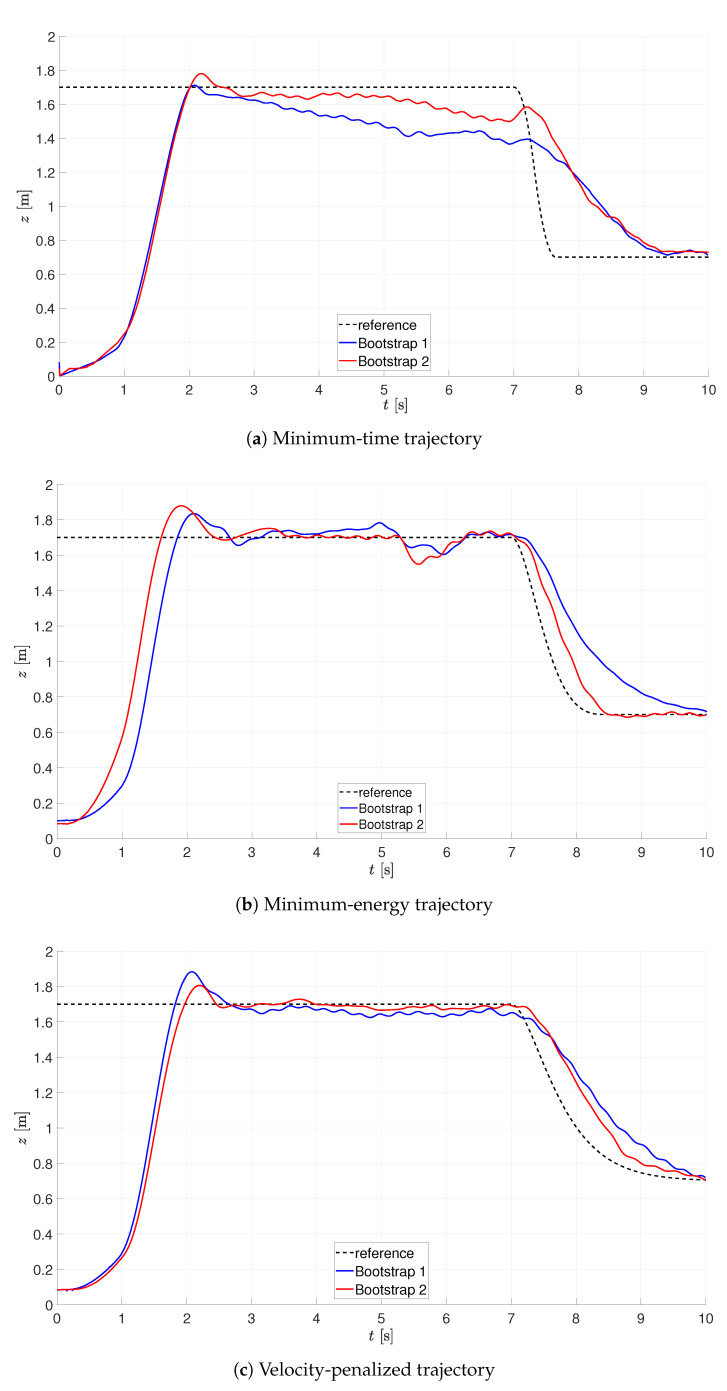
Optimization of landing on the basis of OT readings (tracking).

**Figure 9:**
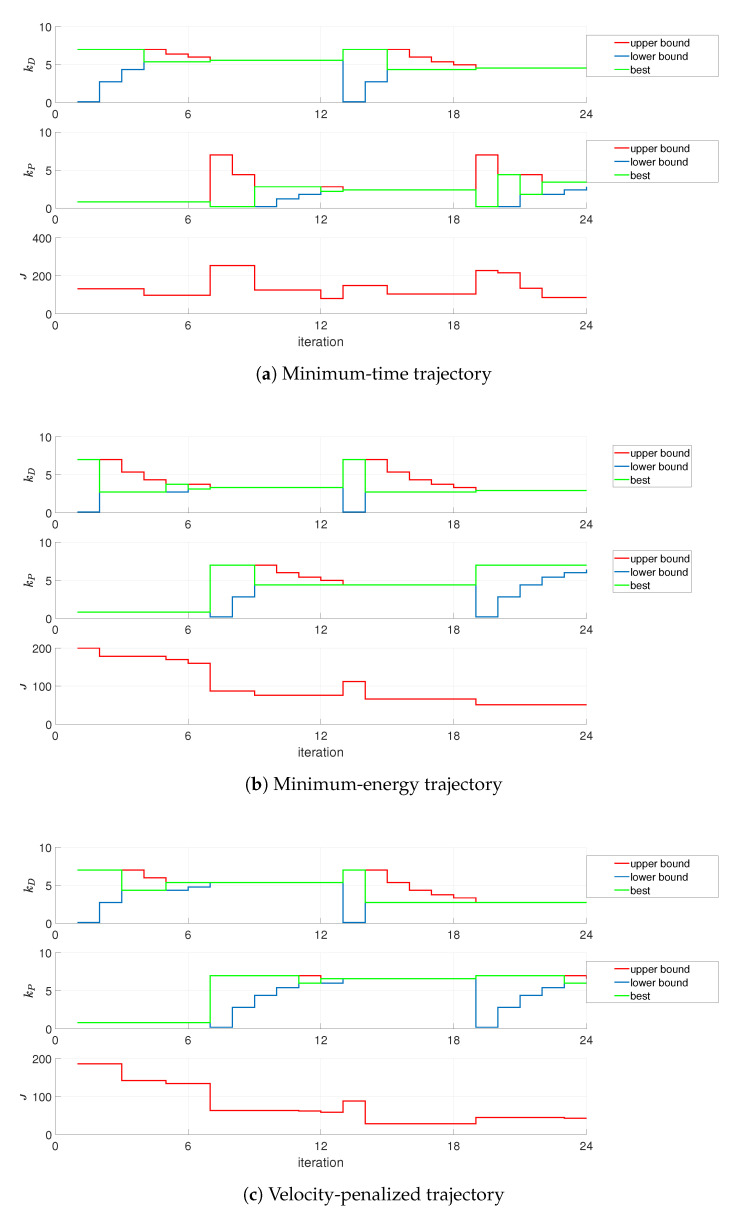
Optimization of landing on the basis of OT readings (gains).

After performing the configuration of the laboratory setup, the tuning experiments were repeated for all the considered trajectories, with the results presented in Figure 8 and Figure 9. As can be seen, the values of performance indices decrease along time, as expected. In all the cases, when the optimization is carried out on the basis of OT readings, it was assumed that: the initial value of $k_{P}=0.8$, the optimal gain $k_{P}$ lies in the range [0.1,7.0], $k_{D}$ lies in the range [0.1,7.0], and ϵ≤0.05 (N=6).

## 8. Experimental Campaign Analysis

**Figure 10:**
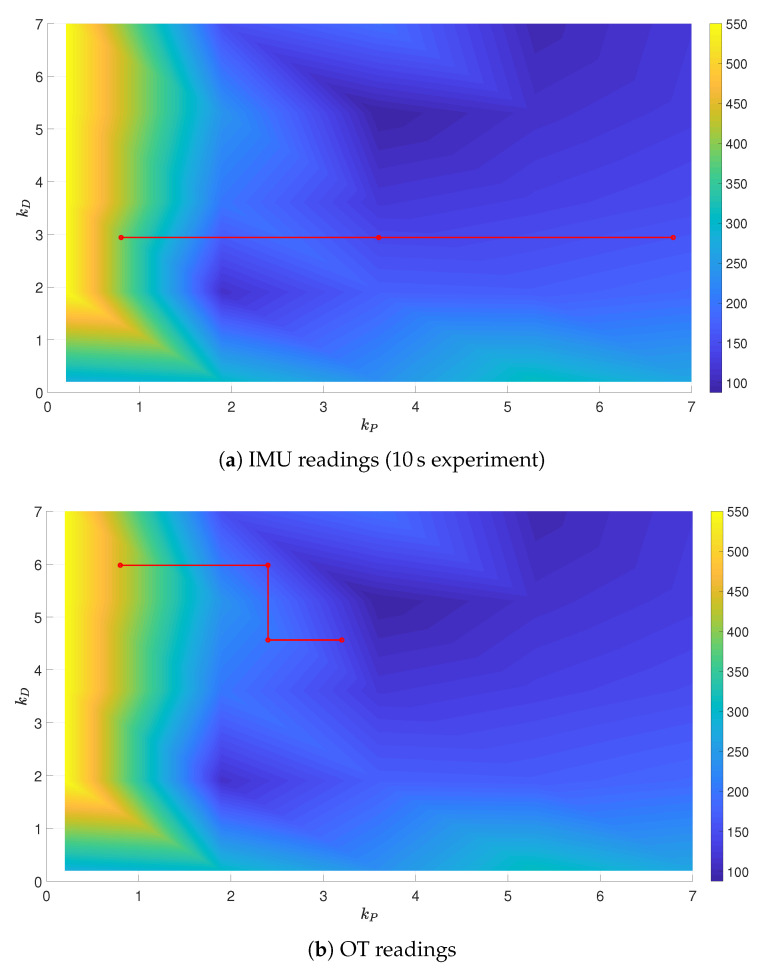
Minimum-time trajectory surface with partial results of tuning.

**Figure 11:**
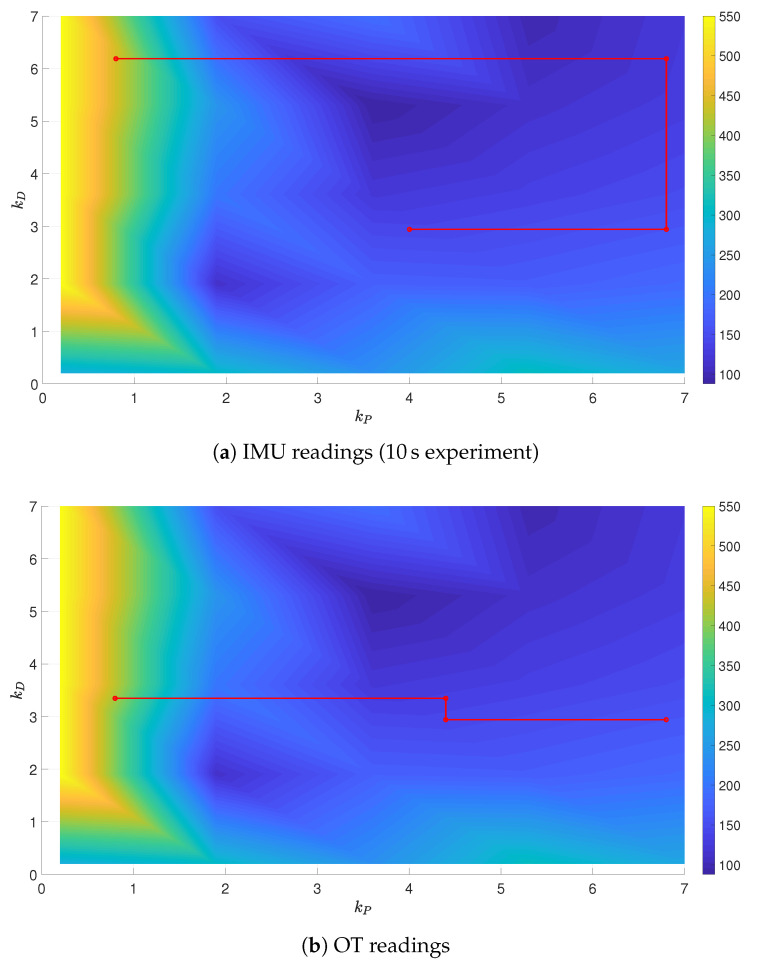
Minimum-energy trajectory surface with partial results of tuning.

**Figure 12:**
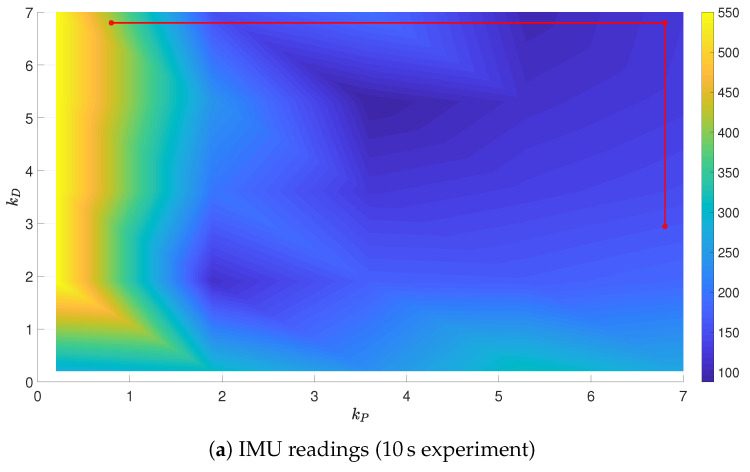

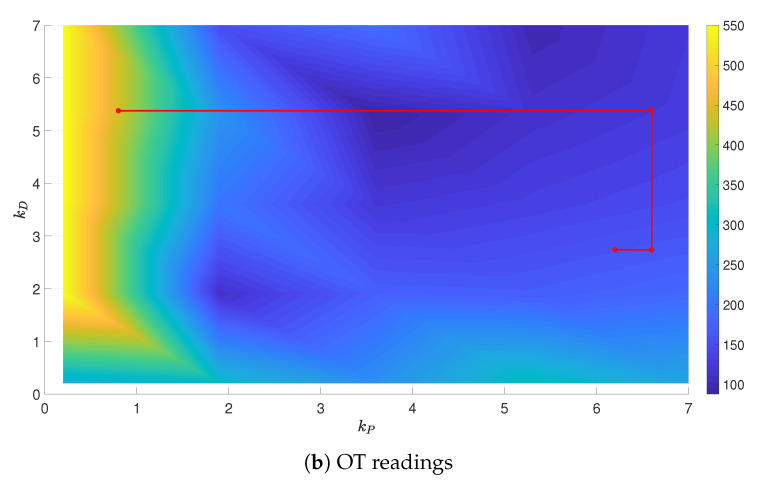
Velocity-penalized trajectory surface with partial results of tuning.

To fully evaluate the results of tuning, a grid of gains $k_{P}$ and $k_{D}$ was generated in size of 30×30. For every point from the grid, a set of experiments was carried out for the specified gains and the selected reference trajectory with the use of the OptiTrack system, with the grid reduced to the size of 5×5. It enabled the authors to present the surfaces of the performance indices $J=f(k_{P},k_{D})$, and the partial solutions to the optimization problem were put on these surfaces, obtained from IMU and OT readings, see Figure 10, Figure 11 and Figure 12. In all the cases, the final gains are close to the minimal values of performance indices depicted in the surfaces. It is to be stressed that in the case of experiments (subfigures (a) and (b)), the performance index values were saturated at 550 in order to clearly present the rest of the values, as low values of $k_{P}$ resulted in infinite growth of the performance index (instability).

The tuning algorithm selects consecutive gain combinations in a way to lower the value of the performance index, though a discrepancy between IMU and OT signal sources can be identified. In the case when the IMU readings are used, the measurement of the actual position of the UAV is impeded, what is mirrored by the tuning results.

**Figure 13:**
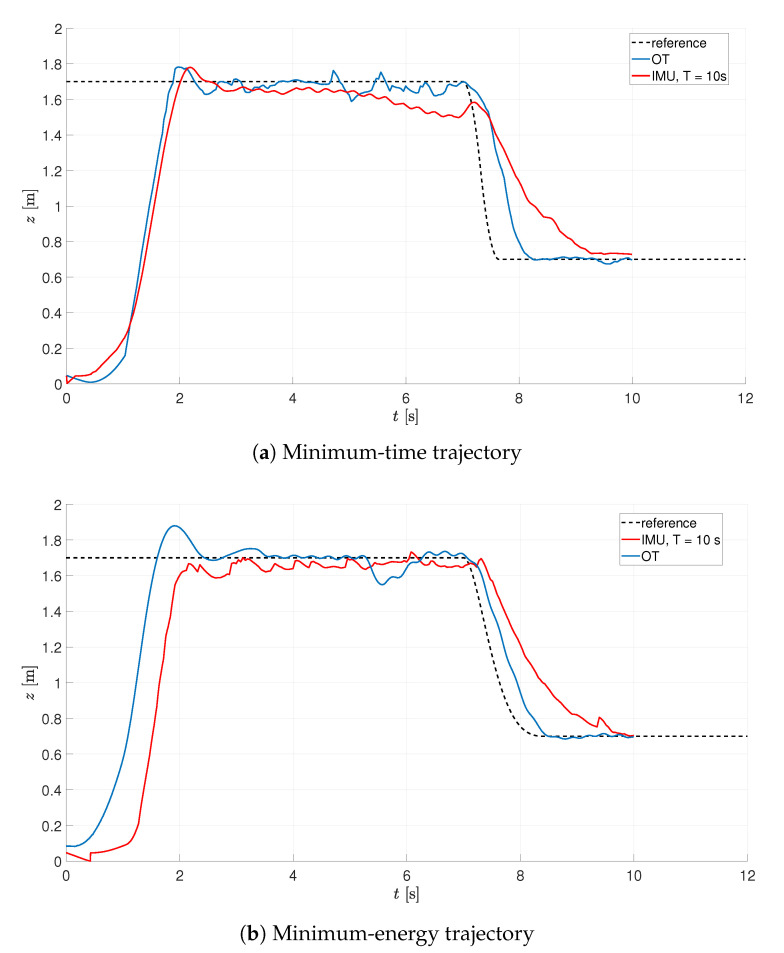

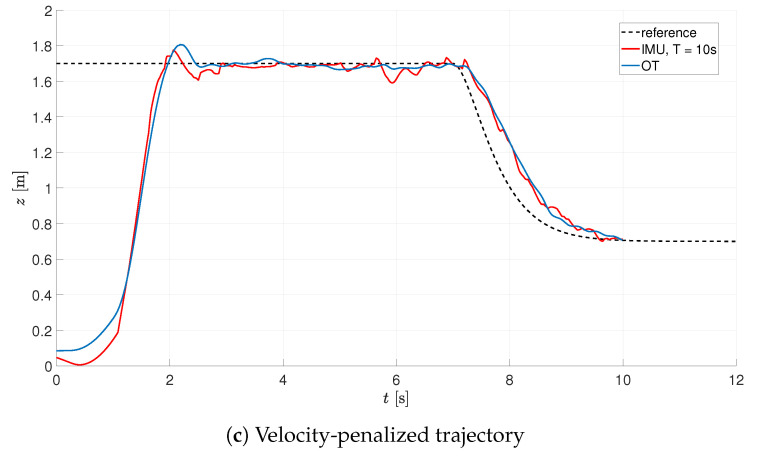
IMU vs. OT performance.

A comparison of final closed-loop responses for the gains obtained on the basis of IMU and OT readings is depicted in Figure 13 and Table 3. Despite the differences in gain values, it is possible to find suitable gain values for both the considered sources of measurements. A neglectful difference between the obtained performance of the system clearly states that it is possible to achieve proper tuning results despite the unavailability of sensors.

The final tuning results were presented in Table 4 to summarize the results in a single place.

## 9. Summary

In this paper, the deployment of the idea of optimization of the landing process of the UAV was shown by means of experiments conducted using the Rolling Spider drone. The obtained results clearly suggest that tuning the altitude controllers is possible in a way as to achieve target-oriented shaping of this control loop, to fit specific dynamics requirements. The paper also showed the procedure to perform this type of tuning in experimental conditions.

The motion capture system was used to precisely measure the position of the Rolling Spider drone, in addition to the IMU-based readings, to give a proper evaluation of the performance of the system. The obtained controller structure proved to be successful inexperiments and in real-world conditions.

In the future work, the authors aim at broadening research towards application in marine-related task of MBZIRC 2023, where the ability to smoothly swop the UAV near the object to be picked, taking performance-related cost criterion into account, should be of value. The research will also be carried out to achieve the ability to optimize the landing procedure in 3D motion, taking the ground effect into account, as well as rejection of disturbances caused by the reverse air flowing subject to near-water positioning.

## Figures and Tables

**Table 1:** Optimization of landing on the basis of IMU readings—10s experiment.

Stage		Trajectory		
		Minimum-Time	Minimum-Energy	Velocity-Penalized
1	$k_{P}$	0.800	0.800	0.800
	$k_{D}$	2.941	6.188	6.797
	*J*	248.025	230.073	239.812
2	$k_{P}$	3.6000	6.800	6.800
	$k_{D}$	2.941	6.188	6.797
	*J*	154.844	135.330	139.708
3	$k_{P}$	3.6000	6.800	6.800
	$k_{D}$	2.941	2.941	2.941
	*J*	139.394	113.712	74.058
4	$k_{P}$	6.800	4.000	6.800
	$k_{D}$	2.941	2.941	2.941
	*J*	114.547	129.908	69.105

**Table 2:** Optimization of landing on the basis of IMU readings—12s experiment.

Stage		Trajectory		
		Minimum-Time	Minimum-Energy	Velocity-Penalized
1	$k_{P}$	0.800	0.800	0.800
	$k_{D}$	3.144	2.941	2.941
	*J*	367.537	413.062	361.338
2	$k_{P}$	6.600	6.800	3.600
	$k_{D}$	3.144	2.941	2.941
	*J*	180.231	362.483	315.960
3	$k_{P}$	6.6000	6.800	3.600
	$k_{D}$	3.144	2.941	1.927
	*J*	364.414	468.594	130.133
4	$k_{P}$	3.000	3.000	3.000
	$k_{D}$	3.144	2.941	1.927
	*J*	302.678	329.685	261.966

**Table 3:** Optimization of landing on the basis of OT readings.

Stage		Trajectory		
		Minimum-Time	Minimum-Energy	Velocity-Penalized
1	$k_{P}$	0.800	0.800	0.800
	$k_{D}$	5.579	3.347	5.377
	*J*	98.137	159.890	134.663
2	$k_{P}$	2.400	4.400	6.600
	$k_{D}$	5.579	3.347	5.377
	*J*	80.3492	76.008	58.869
3	$k_{P}$	2.400	4.400	6.600
	$k_{D}$	4.565	2.941	2.738
	*J*	104.228	65.839	28.500
4	$k_{P}$	3.200	6.800	6.200
	$k_{D}$	4.565	2.941	2.738
	*J*	85.860	51.582	43.027

**Table 4:** Comparison of the obtained optimal gains.

Tuning		Trajectory		
		Minimum-Time	Minimum-Energy	Velocity-Penalized
IMU 10s	$k_{P}$	6.800	4.000	6.800
	$k_{D}$	2.941	2.941	2.941
	*J*	114.547	129.908	69.105
OT	$k_{P}$	3.200	6.800	6.200
	$k_{D}$	4.565	2.941	2.738
	*J*	85.860	51.582	43.027

**Figure A1:**
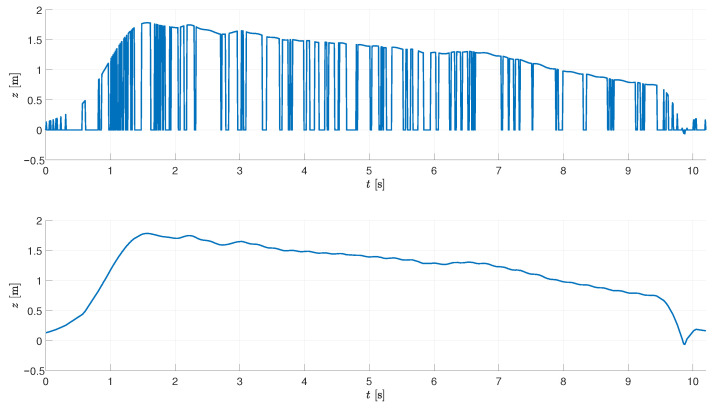
Interpolated data from the OptiTrack system.

**Figure A2:**
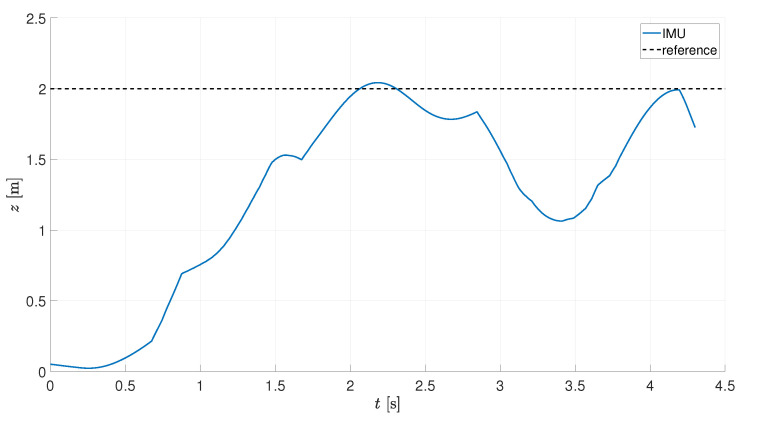
Data from the IMU of the Rolling Spider (reference signal vs. IMU measurements).

## Data Availability

Data available on request due to restrictions.

## Appendix A. Configuration Tweaks

In the case of occlusion of a marker under a MoCap system, the OptiTrack assumed the UAV was at the point (0,0,0), what could potentially lead to performance evaluation deterioration, as the values of optimization performance indices were amended, leading to improper tuning and optimization. The missing data was replaced with their first-order interpolated estimates, with the use of a script given in Listing A1, and the graphical results presented in Figure A1.

Listing A1 Script interpolating data obtained from the OptiTrack system between the first available left and first available right samples **function** [yout] = interpolateFirstOrder(u)
 			
y = **zeros**(**length**(u),1);
maxIndex = **length**(u);
%%*find first nonzero*
nonZero = **find** (u > 0.0);
**if** (**isempty** (nonZero))
     yout = u;
**else**
     y(1) = u(nonZero(1));
     i = 2;
     **while** i < **length** (u)
	  **if** (u(i) ~= 0)
	       y(i) = u(i);
	       i = i + 1;
	  **else**
               leftValue = u(i − 1)
               rightValue = 0.0;
               shift = 0;
	       **while** (i + shift − 1) < maxIndex && rightValue == 0.0
		     shift = shift + 1;
		     rightValue = u(i + shift);
		 end
		 if (rightValue == 0.0)
	            rightValue = leftValue;
		 **end**
		 step = (rightValue − leftValue)/(shift + 1);
			
		 **for** j = 0: shift
		     y (i + j) = leftValue + ( j + 1) * (step);
		 **end**
		 i = i + shift;
	     end
	 end
	 if (u(**end**) == 0.0)
	     y (**end**) = y (**end** − 1);
	 **end**
	 yout = y;
end
 
end

To verify the solutions presented in the current paper, experiments on the real Rolling Spider platform were conducted, with the use of Linux Ubuntu 14.04 LTS system. It was necessary to use this particular version, as the *Simulink Support Package for Parrot Minidrones* used a Personal Area Network (PAN) connection initialized by the *BlueZ Bluetooth PAN daemon* software not supported by Ubuntu 16.04 LTS system. The second step was the update of the original firmware of the Rolling Spider, which is usually done once only, and allows one to pair a computer with the UAV, and to get access to the MAC address of the drone, to establish connection, see [34].

The Bluetooth 4.0 standard equipment or newer is necessary to proceed to the experimental stage, what was done with the use of the DroneConnect.sh script, from the drone manufacturer. Once the PAN type connection is established, the Rolling Spider is visible under the IP 192.168.1.2, and it is accessible via Telnet protocol.

During the tests, numerous altitude estimation problems were encountered, whenever Inertial Measurement Unit (IMU) of the Rolling Spider was used. It was quite often when the drone ascended to 2m to lose stability and to increase its altitude with no control. The examination of the flight data leads to the plot visible in Figure A2, where it is shown that despite flying to a 2.5m height, the IMU readings show the expected altitude is not reached.

It was crucial to avoid this problem, as the correct estimation of the altitude was necessary for successful optimization of the landing process. After multiple tests, it was identified that the actions of IMU resulted from the issues listed below:

- the ultrasound sensor has a limited range, and flying above 2m makes its reliable use impossible (due to large uncertainty of the measurements)—as the result, the starting altitude was decreased to 1.7m in all tests;
- the ultrasound sensor produces a cone-shaped waves, and there should be no obstacles within this area, thus the experiments need to be carried out in the area of at least 3×3m in size;
- using the barometer to estimate altitude requires one to define the current air density, as well as its shear viscosity—by default $\rho =1.184\;\frac{\mathrm{kg}}{m^{3}}$ and $\upsilon =15\times 10^{6}\;\frac{m^{2}}{s}$, respectively, what corresponds to the atmospheric pressure of 1013hPa at the temperature of $20{\;}^{\circ }C$; as the tests were conducted at the temperature of $30{\;}^{\circ }C$, it was assumed that $\rho =1.165\;\frac{\mathrm{kg}}{m^{3}}$ and $\upsilon =16\times 10^{6}\;\frac{m^{2}}{s}$ [35];
- position estimate of the UAV is obtained from its on-board 0.3Mpx camera, and the tests should be carried out using a solid ground, with the preferably well-visible pattern; sticking e.g., a white insulation tape to the ground strongly improves the IMU readings, and admits better stabilization during in-flight conditions.

Some flight safety tweaks also had to be introduced to the software, as it cuts off the power from the motors in case of a low battery state, or whenever the acceleration is exceeded in any of the axes. During the optimizaton of the landing process, it was quite common that when the minimum-time scenario was adopted, the admissible acceleration was exceeded, leading to cutting of the power supplies (see the remaining part of the paper for details on landing scenarios). The admissible values are defined in the file MIT_MatlabToolbox/trunk/embcode/rsedu_control.c, and changing them (see the Listing A2 and Listing A3) enables the structure to have greater acceleration. It was also found necessary to alter the MIN_BATTTTAKEOFF value, down to the level of 30% enabling take off procedure during the optimizaton procedure, what positively affected the time available for tests.

Listing A2 Safety parameters rsedu_control.c. **static float** MAX_ACCELL = 10.0; //= *6.0*;
**static float** MAX_DELTADXY = 6.0;
**static float** MAX_RANGE = 10.0;
**static float** MIN_BATTTAKEOFF = 30.0; //= *50.0*;
**static float** MIN_BATT = 30.0;
**static int**   MAX_noOF = 50;

Listing A3 Excessive acceleration testrsedu_control.c.
//*NOSAFETY*  *disabled and after* *takeoff-cycles* 
**if**( (!FEAT_NOSAFETY) && (counter>(calibCycles + takeoffCycles)))
crash_detected = (fabs(in->HAL_acc_SI.x) > MAX_ACCELL)||...
 (fabs(in->HAL_acc_SI.y) > MAX_ACCELL) || (in->HAL_acc_SI.z >
  MAX_ACCELL);
